# Gastrointestinal stromal tumor masquerading as a lung neoplasm. A case presentation and literature review

**DOI:** 10.1186/1749-8090-3-31

**Published:** 2008-05-21

**Authors:** S Papaspyros, K Papagiannopoulos

**Affiliations:** 1 Department of Thoracic Surgery, St. James University Hospital, Beckett Street, Leeds LS9 7TF, UK

## Abstract

Gastrointestinal stromal tumors (GISTs) are rare neoplasms of the gastrointestinal tract. Their incidence in the esophagus is 1%–3%. Never has a GIST been documented to directly invade the lung. We report a primary esophageal GIST with direct invasion into the lung parenchyma, presenting predominantly with respiratory symptoms. We include a retrospective literature review. Although the principle 'common things are common' usually guides our everyday clinical practice, this case emphasizes that rare entities can mimic common pathologies and underlines the importance of having a clearly defined differential diagnostic list which should be meticulously scrutinized.

## Introduction

Gastro-Intestinal Stromal Tumors (GISTs) belong to the group of gastrointestinal mesenchymal tumors (GIMTs). These include myogenic tumors (leiomyomas or leiomyosarcomas), neurogenic tumors (schwannomas) and GISTs. They arise from the gastrointestinal (GI) wall, and they are distributed from the esophagus to the rectum. [1-4]

These are now defined as spindle cell, epithelioid or occasionally pleiomorphic mesenchymal tumors that express c-kit protein immunopositivity (CD117), which is their major diagnostic criterion. [2,3,5]

Although generally rare, they are the most common mesenchymal neoplasms of the GI tract, and encompass most tumors previously classified as gastric and intestinal smooth muscle or neural cell tumors [1,2].

## Case report

A 58 year old lady was referred by her General Practitioner (GP) after presenting with a 3 day history of moderate left sided pleuritic chest pain radiating to the left shoulder, low grade pyrexia and frontal headaches. She experienced no cough, hemoptysis, or shortness of breath but had recurrent episodes of upper respiratory tract infections in the past five months. There was no significant past medical history and she was a lifetime non smoker.

**Figure 1:**
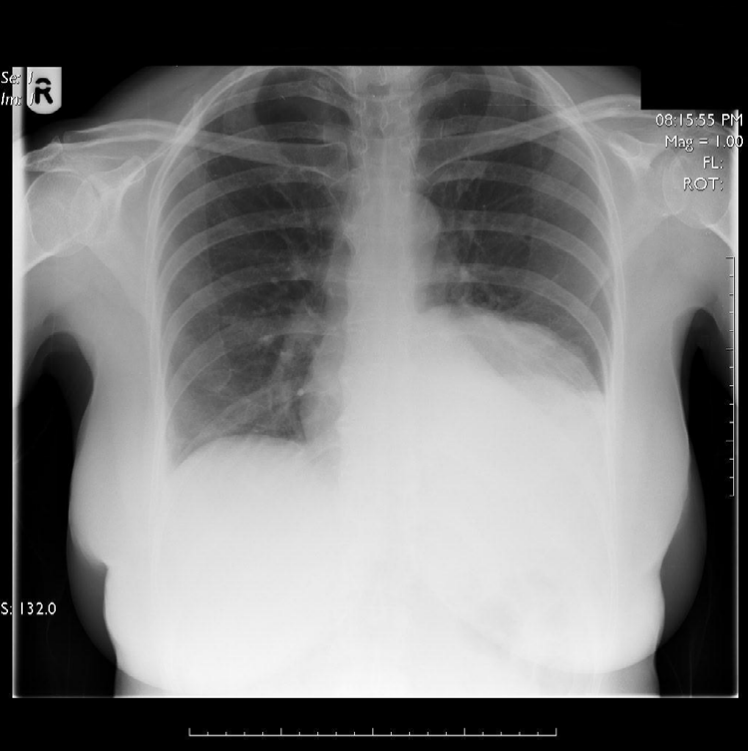
Chest radiograph showing a raised left hemi-diaphragm and volume loss in the left lower lobe.

Clinical examination revealed left lower lobe crackles. Routine blood tests revealed a White Cell Count of 11.82 10^9^/lt and a C-Reactive protein (CRP) of 164 mg/lt. The chest radiograph (CxR) demonstrated a raised left hemi diaphragm with volume loss in the left lower lobe (Fig 1).

**Figure 2:**
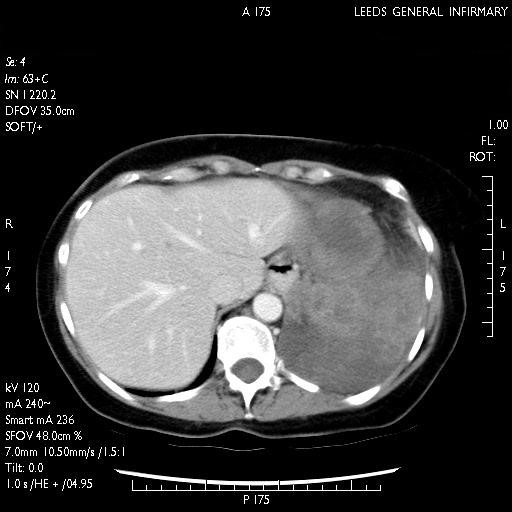
CT scan depicting a well circumscribed tumor measuring 10 cm antero-posteriorly, 14 cm transversely and 9 cm cranio-caudally enveloping the descending aorta and extending into the posterior mediastinum.

Due to the equivocal findings on the CxR, she underwent a Computerized Tomography (CT) (Fig 2,) of her thorax which revealed a large necrotic mass within the left lower lobe extending into the posterior mediastinum, surrounding the aorta and in close contact with the distal esophagus. It extended to the gastric fundus through extensive invasion of the left hemi-diaphragm. No mediastinal lymphadenopathy was reported.

A CT guided biopsy was performed and the histology showed a spindle cell tumor. Immunohistochemistry revealed the tumor cells to be positive for CD 34, h-caldesmon (muscle marker) and focally positive for epithelial membrane antigen. The possibility of a 'solitary fibrous tumor' was raised as the clinical history indicated that this was primarily a chest tumor. She was subsequently referred to our team.

At thoracotomy the tumor was invading the left lower lobe but large part of it was descending in the posterior mediastinum and through the hiatus. The initial incision was extended to a left thoracolaparotomy and the mass was resected en block performing a left lower lobe lobectomy, a distal esophagectomy, partial gastrectomy and diaphragmatic resection with primary esophagogastric anastomosis and diaphragmatic reconstruction.

**Figure 3:**
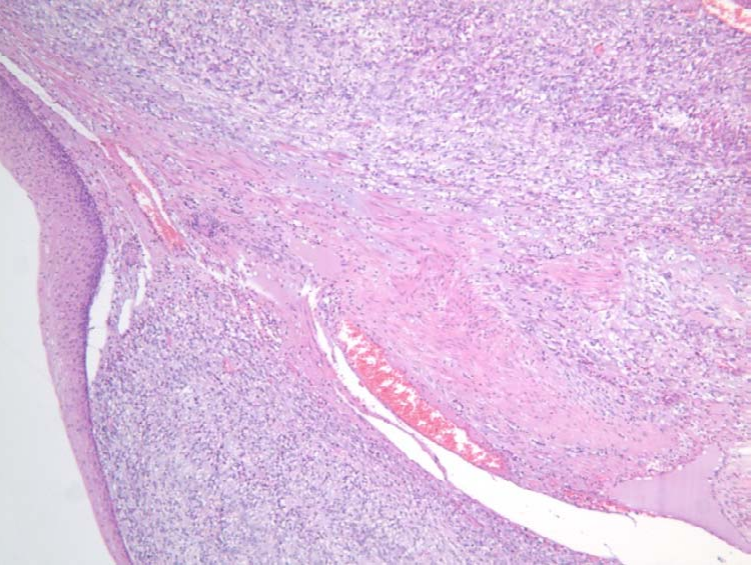
Slide depicting H & E stain of the GIST within the esophageal wall (Magnification power 5×).

On histopathological examination the tumor was predominantly composed of spindle shaped cells, with oval nuclei and eosiniphilic cytoplasm. Focal myxoid change was present. There was some nuclear pleomorphism and in areas the mitotic count was more than 5/50 high power field (HPF). Although the tumor was predominantly within the lung parenchyma, it was seen to be arising from the esophageal wall, where it extended into the lamina propria and attenuated the overlying esophageal squamous epithelium (figure 3).

**Figure 4:**
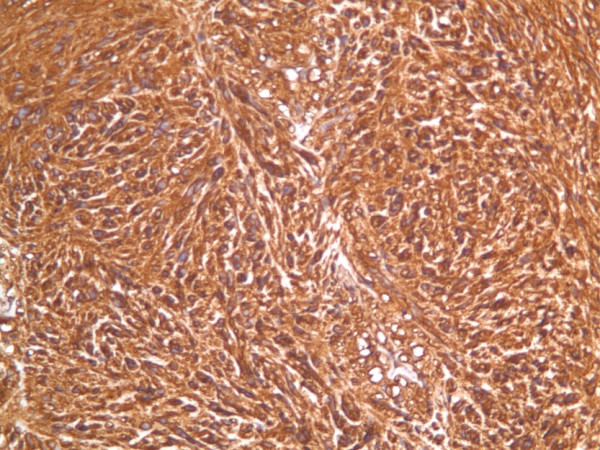
Slide depicting c-KIT (CD 117) stain of the GIST with diffuse positivity (Magnification power 20×).

On immunohistochemistry, the tumor cells revealed strong diffuse positivity for CD 34 and CD 117 (c-kit) (figure 4). The cells were focally positive for the muscle markers including h-caldesmon, desmin, actin and myoglobin. Focal positivity was also seen with epithelial membrane antigen and cytokeratin (MNF 116). The tumor was negative for S 100 protein CD 99, Thyroid transcription factor (TTF 2) and calretinin.

On the basis of the above findings, the tumor was classified as a gastrointestinal stromal tumor arising from the esophageal wall and extending into the lung parenchyma. As the tumor measured more than 10 cm in size, with a mitotic count over 5/50 HPF extending into the lung parenchyma, it was categorized as a high risk GIST.

Following post-operative recovery, the patient was referred to the oncology team for assessment regarding adjuvant therapy.

## Discussion

### Incidence

According to the largest epidemiologic analysis done so far, which included 1458 recorded cases [6], as well as studies by Miettinen et al [2], GISTs typically present in adults over 40–50 years and only exceptionally in children [2,7]. Their incidence according to anatomic location varies among different studies and ranges between 51%–70% in the stomach, 25%–36% in the small intestine, 5%–7% in the colon, rectum and appendix, and 1%–3% in the esophagus. Primary GISTs can be found in the omentum, mesentery or retroperitoneum, unrelated to the tubular GI tract, but most in these sites are metastatic from gastric or intestinal primaries [2,4,6,8-10].

### Histologic features

GISTs can exhibit either a spindle, epithelioid or mixed cytomorphology. Spindle cell GISTs are usually arranged in fascicles, while epithelioid GISTs are arranged in sheets or nests. Mitotic activity is variable. Necrosis can be present. Nuclear pleomorphism is rare, usually focal.

### Immunohistochemical features and histogenesis

According to a concensus approach on the diagnosis of GISTs [5] the term "GIST" should only apply to neoplasms displaying KIT (CD117) immunopositivity with very rare exceptions. Recently, some GISTS without the *KIT *mutation have been found to express a mutation in another tyrosine kinase receptor gene, the *PDGFRa *gene. It is important to mention that normal Kit-positive cells in abdominal soft tissues include mast cells present in the wall of the GI tract and the interstitial cells of Cajal (intestinal pacemakers) present around the myenteric plexus [3]. Although the origin of GISTs is not fully understood, their association with the Cajal cells suggests that these cell subsets could represent a multipotential stem-cell like population, which is the logical candidate for GIST histogenesis [1]. Positivity for nestin (90%–100%) and CD34 (70%) are also characteristic but not specific [2].

### Prognostic factors

Amongst histologic criteria the most important prognostic factor is mitotic index [1,2,11]. Other less important criteria are high cellularity, marked pleiomorphism and presence of histological necrosis [1]. Among clinical criteria the size of the tumor is the most important prognostic factor [2,8]. Other less important clinical criteria are macroscopic invasion into surrounding structures, metastasis at diagnosis, peritoneal dissemination, tumor rupture at surgery and incomplete resection [1,5,8]. However, low mitotic index and small size do not absolutely guarantee a benign clinical course. Therefore, instead of classifying lesions as either benign or malignant, current guidelines categorise GISTs as low, intermediate and high risk based on size and mitotic index[12]. High risk GISTs have an increased potential for diffuse intra-abdominal spread and liver metastasis, which are the two most common modes of dissemination [3,8]. Distant metastasis to other sites, especially bone and lung, are relatively rare [3,8].

### Clinical presentation of GISTs

The most frequent clinical manifestations are occult gastrointestinal bleeding, pain, dyspepsia, fatigue associated with anemia, palpable mass, perforation or rarely obstruction [13]. In a study of 17 esophageal GISTs [4], seven presented with dysphagia, two had cough, one had gastrointestinaI bleeding, and two had weight loss. There has been no report of a direct local invasion of a GIST tumor to the lung and only in one study is the lung mentioned in association with a GIST tumor and only as metastatic site from an intestinal primary [8].

### Management-Survival

Complete surgical resection remains the standard treatment for primary, non metastatic GISTs. The tumor is often fragile, with haemorrhage or necrosis, and it may have a pseudocapsule. Meticulous surgical technique is necessary to remove the tumor en-bloc and avoid intraoperative rupture which is associated with poor prognosis [11,13]. A wide resection margin is not needed, however local peritoneal seeding is common and a local peritonectomy should be performed when feasible [13]. Lymph node metastases are rare hence routine regional lymphadenectomy is not recommended [13,14]. Given the potential malignant behavior of benign appearing GISTs, at least one group of investigators believes that all should be classified as malignant tumors on a low-to-high grading scale rather than on a benign-versus-malignant basis [15]. Malignant GISTs are highly refractory to conventional chemotherapy and radiotherapy [6,8]. Five year survival following complete surgical resection varies ranging between 35%–60% [13].

Since 1988 the treatment of GISTs has changed dramatically after discovering that the majority of these tumors have oncogenic mutations of the KIT receptor tyrosine kinase [16]. As well as being a useful diagnostic marker, KIT became an excellent therapeutic target [17]. Imatinib mesylate, is a molecule that selectively inhibits the enzymatic activity of the ABL and BCR-ABL fusion protein, PDGF-receptor and KIT tyrosine kinases. Imatinib mesylate inhibits the mutated KIT receptor leading to apoptosis and decrease in proliferation of tumor cells [17,18]. Several trials are still ongoing but preliminary results support the effectiveness and safety of its use in unresectable, recurrent and metastatic tumors and has been reviewed systematically [14].

### Comments

Gastrointestinal stromal tumors are rare in the esophagus and have never been documented before this case report to directly invade the lung. Despite the limited experience, complete surgical resection is indispensable and adjuvant imatinib mesylate therapy is recommended in cases of high risk. Therefore surgeons, physicians and especially pathologists should keep in mind the possibility of GISTs when a lesion mainly occupying the lung is shown radiologically to involve the esophagus or stomach. In such cases immunohistochemical investigations should include CD117, CD34 and muscle markers so that an accurate diagnosis is reached, and the appropriate treatment instituted.
